# The complete mitochondrial genome of *Platycheirus albimanus* (Diptera: Syrphidae: Syrphinae) and phylogenetic analysis of the Syrphidae

**DOI:** 10.1080/23802359.2021.1872455

**Published:** 2021-02-11

**Authors:** Yan Yan, Li Hu

**Affiliations:** School of Biological Science and Engineering, Shaanxi University of Technology, Hanzhong, Shannxi, China

**Keywords:** Hoverfly, mitogenome, phylogeny, *Platycheirus albimanus*

## Abstract

The complete mitochondrial genome of *Platycheirus albimanus* (Fabricius, 1781) was sequenced. The length of the mitogenome is 16,648 bp and consists of 37 genes including 22 transfer RNA (tRNAs), 13 protein-coding (PCGs) and 2 ribosomal RNA (rRNAs). The 13 PCGs initiate with the start codon ATN, except for *COX1* and *ATP6* which use TTG. All of the PCGs ended with TAA, apart from *ND4* and *ND4L* terminated by incomplete T––. The ML tree based on complete mitogenomes from 25 species (22 Syrphidae and 3 outgroup taxa) suggests that the tribe Melanostomini is more closely related to the Syrphini. The phylogenetic analysis supports the monophyly of Syrphinae, and the paraphyly of the Eristalinae. This mitogenome information for *P. albimanus* could facilitate future studies of evolutionarily related insects.

*Platycheirus albimanus* (Fabricius, 1781), the common hoverfly, is classified to the subfamily Syrphinae (Diptera: Syrphidae). It is a flower-visiting insect found in grass and herb vegetation. *Platycheirus albimanus* always feed on aphids (Young et al. 2016). This species is characterized by the following features: evenly black body, medium-sized with length of 6.2–9.6 mm, gray spots on abdominal tergites, and the obviously widened tibia and tarsus of the front legs (Huo et al. 2007; Huang and Cheng 2012).

To date, 21 complete mitochondrial genomes (mitogenomes) have been registered in the GenBank (https://www.ncbi.nlm.nih.gov/) for the Syrphidae (Cameron et al. 2007; Junqueira et al. 2016; Pu et al. 2017; Li et al. 2017; Li and Li 2019; Li 2019; Sonet et al. 2019; Chen et al. 2020; Liu, Song, et al. 2020; Liu, Wang, et al. 2020; Yan et al. 2020; Zhao and Li 2020). In this study, we sequenced and assembled the complete mitogenome of *P. albimanus.* A phylogenetic analysis was performed using all known complete mitogenomes of Syrphidae to better understand the phylogenetic relationships of this species in the family.

The specimens of *P. albimanus* were collected from the campus of Shaanxi University of Technology (107°02′ E, 33°04′ N) in the Hanzhong City of Shaanxi Province on March 2019. The specimens were immediately preserved in absolute ethanol and frozen at −20 °C and kept at the Museum of Zoology and Botany, Shaanxi University of Technology, Hanzhong, China (SUHC) under the accession number 201901-38.

Genomic DNA of *P. albimanus* was extracted using the TIANamp Genomic DNA kit (Tiangen, Beijing, China). The mitogenome was sequenced using the Illumina NovaSeq 6000 platform, and assembled and annotated with Geneious Prime (Kearse et al. 2012). The tRNAs were predicted by ARWEN v1.2 (Laslett and Canback 2008), and the rRNAs and control region were identified by alignment with homologous genes of previously determined mitogenomes of Syrphidae.

The complete mitogenome of *P. albimanus* is 16,648 bp (GenBank No. MT622646) in length, the mitogenome structure is the same as that of most insects of Diptera, including 22 tRNAs, 13 PCGs, 2 rRNAs and control region, of which 23 genes are located on the J-strand and 14 genes are encoded in N-strand. Twenty-one intergenic spaces and 7 gene overlaps were identified, the length of them varying from 1 to 20 bp, and from 1 to 13 bp, respectively.

The nucleotide composition of *P. albimanus* was significantly biased toward A and T (40.7% of A; 40.3% of T; 10.7% of C; 8.2% of G), with an AT bias of 81%. With the exception of *COX1* and *ATP6* which initiated with the TTG codon, all other PCGs started with ATN. The *ND4* and *ND4L* terminated with an incomplete T— codon, while the remaining PCGs stopped with TAA. The tRNA genes range from 65 bp (*tRNA-Arg* and *tRNA-Glu*) to 72 bp (*tRNA-Val*).

A phylogenetic tree was constructed based on the complete mitogenome sequences from 22 Syrphidae and three outgroups (*Nemopoda mamaevi*, *Pachycerina decemlineata*, *Cestrotus liui*) (https://www.ncbi.nlm.nih.gov/) using the Maximum-Likelihood (ML) substitution model and Kimura 2-parameter with 500 bootstrap replicates with the software MEGA7 (Kumar et al. 2016). The result shows that *P. albimanus* was clustered in the Syrphinae clade and sister to *Melanostoma* in the Melanostomini. The Melanostomini and Syrphini are resolved in a fully supported clade in the Syrphidae (Figure 1). The monophyly of the subfamily Syrphinae is supported, and agrees with previous studies (Li 2019; Li and Li 2019; Zhao and Li 2020). However, the Eristalinae is paraphyletic in this analysis, which is similar to the findings of Zhao and Li (2020).

**Figure 1.**
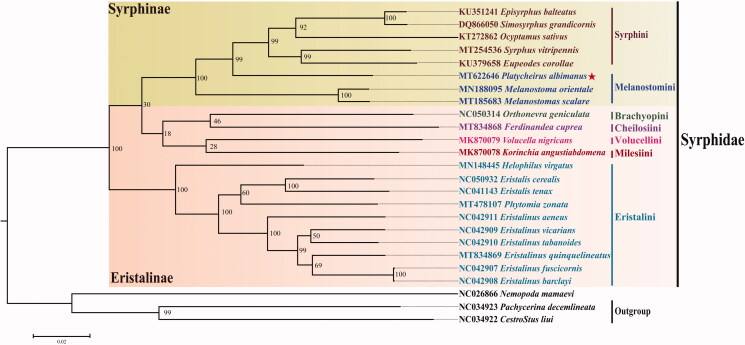
Maximum-Likelihood tree of *Platycheirus albimanus* and Syrphidae based on 25 complete mitogenomes using Maxumum likelihood (ML). Numbers at the nodes represent bootstrap support values based on 500 replicates and indicates the new sequence in this study.

## Data Availability

Mitogenome data supporting this study are openly available in GenBank at nucleotide database, https://www.ncbi.nlm.nih.gov/nuccore/MT622646, Associated BioProject, https://www.ncbi.nlm.nih.gov/bioproject/687806, BioSample accession number at https://www.ncbi.nlm.nih.gov/biosample/SAMN17154901 and Sequence Read Archive at https://www.ncbi.nlm.nih.gov/sra/SRR13306841.
